# Self-Regulatory Strategy Use, Efficacy, and Strategy-Situation-Fit in Self-Control Conflicts of Initiation, Persistence, and Inhibition

**DOI:** 10.1177/08902070221150478

**Published:** 2023-01-17

**Authors:** Mario Wenzel, Sebastian Bürgler, Veronika Brandstätter, Antonia Kreibich, Marie Hennecke

**Affiliations:** 1 9182 Johannes Gutenberg Universität Mainz, Mainz, Germany; 2 14312 Universität Siegen, Siegen, Germany; 3 30963 Universität Zürich, Zurich, Switzerland

**Keywords:** self-regulation, self-control, strategies, strategy-situation fit, regulatory flexibility

## Abstract

Self-control is the ability to (1) initiate, and (2) persist in boring, difficult or disliked activity, and to (3) inhibit impulses to act. We explored the self-regulatory strategies that people use for these three types of self-control conflicts and their subjective efficacy as a function of conflict type. In addition, we hypothesized that people who more frequently create strategy-situation fit by tying strategies to the conflict types they are effective for, are more successful at self-control. A pilot study identified 22 different self-regulatory strategies that could be used for more than one type of self-control conflict. We then used a large data set from two pooled experience sampling datasets (*n* = 14,067 reported self-control conflicts) to quantify these strategies’ popularity and subjective efficacy in daily life. Eight strategies were positively and three negatively associated with subjective self-regulatory success but subjective efficacy often depended on type of conflict: Some strategies were effective and some maladaptive only for some types of self-control conflicts. Individuals who created strategy-situation fit for some strategies also reported greater self-regulatory success, as hypothesized. We discuss regulatory flexibility as a crucial component of good self-control.

In the famous “marshmallow test,” 4-year old children were confronted with what is now an iconic self-control conflict: They could either eat a marshmallow that was placed in front of them right away or they could wait until the experimenter returned and provided them with an additional second marshmallow in exchange for their patience (Mischel et al., 1989). Patient children were often observed using certain self-regulatory strategies that appeared to help them endure the waiting time. Indeed, experimental research supported the observation that distraction is a useful strategy for increasing the ability to delay gratification in this situation (Mischel et al., 1972).

In adults’ everyday lives, resisting the lure of one marshmallow to attain an additional one may be less of a concern. Typical scenarios requiring self-control in adulthood involve resisting the lure of junk food, getting up from the couch to exercise, or staying on the treadmill at the gym for a sufficient duration. These scenarios have in common with the famous delay-of-gratification paradigm described above that a smaller and proximal reward (e.g., delicious junk food and resting) is at odds with the pursuit of a larger and more remote reward or goal (e.g., being physically fit) (Fujita, 2011; Hoch & Loewenstein, 1991; Mischel et al., 1989). These scenarios *differ* from each other*,* however, in a way that has received only little attention so far (but see De Boer et al., 2011; De Ridder et al., 2011; Hoyle & Davisson, 2016): The first scenario, resisting junk food, requires the *inhibition* of an undesired response to a temptation, just as resisting a marshmallow does. Such a self-control conflict of inhibition occurs when a person needs to override “a pull toward goal-inconsistent behavior” (Hoyle & Davisson, 2016, p. 399). The second exemplary scenario, getting up from the couch to exercise, in contrast, is better characterized as requiring the *initiation* of a desired goal-directed behavior. In a self-control conflict of initiation, a person experiences a “pull for inaction when pursuit of an active goal requires action” (Hoyle & Davisson, 2016, p. 399). Finally, the third exemplary scenario, continuing an effortful exercise, requires *persistence* in a desired goal-directed behavior. A self-control conflict of persistence occurs when a person needs “to continue following initiation despite the pull to stop when a self-control challenge is on-going” (Hoyle & Davisson, 2016, p. 399). In line with these differences, Carver (2019, p. 477) has defined self-control as the ability to “override impulses to act”, “make oneself initiate,” and “persist in boring, difficult, or disliked activity.”

Whereas all three types of self-control conflicts involve dilemmas between a current goal and competing impulses, habits, or desires, little is known about differences (and commonalities) in the *types of self-regulatory strategies* people may use depending on the type of conflict and differences (and commonalities) in strategy *efficacy* as a function of self-control conflict type. Exploring these are aims of the current investigation. In addition, we investigated the hypothesis that people who more frequently create *strategy-situation fit* by tying strategies to the conflict types they are effective for, are more successful in resolving their self-control conflicts. While this aspect of regulatory flexibility has been studied in how people deal with their emotions, our study is the first to attest to its importance in the context of daily self-control conflicts.

## Self-Regulatory Strategies

Self-regulatory strategies can, like emotion regulation strategies, be distinguished according to the processes they rely on and according to where in the unfolding event, the emergence of an emotion or a self-control conflict, they exert their influence (Duckworth et al., 2016). Strategies relying on *situation selection* and *situation modification* can be used to prevent self-control conflicts from occurring in the first place. A dieter might, for example, avoid walking by his favorite bakery to not become tempted by the smell of delicious pastry (situation selection). Strategies relying on *attentional deployment* involve directing one’s attention to those aspects of a situation that advance self-control and away from distractions, temptations, or any other aspects that may undermine it. A runner at the gym might, for example, focus their attention on the rhythm of their pace to distract themselves from his aching muscles. Strategies relying on processes of *cognitive change* can be deployed to change one’s own interpretation, construal, or understanding of the situation in which a self-control conflict occurs. A student may reframe solving a difficult chemistry exercise as a means to making his dream of becoming a physician come true. Lastly, strategies relying on processes of *response modulation* target the unwanted behavioral outcome itself through the suppression of unwanted or the forceful execution of goal-directed behaviors (Duckworth et al., 2016). A dieter may suppress the urge to eat a second piece of cake and a runner at the gym might force themselves to just keep going.

Situation selection, situation modification, attentional deployment, cognitive change, and response modulation are probably best understood as relatively broad categories of strategies with different and diverse lower-level strategies within them (Hennecke et al., 2019). Situations may, for example, be modified in various, sometimes very different ways. When confronted with a temptation, a person may, for example, move the temptation to a different location to not be confronted with it any further. When needing to continue with an unpleasant activity, a person may ask another person for support or add some positive external stimulation, for example, music, to make the activity more bearable. Cognitive change may also look very different depending on what the person tries to think about. For example, trying to cognitively reappraise a piece of cake as an unhealthy compound of fat and sugar may have different effects on self-control than thinking about the positive consequences of not eating it, for example, a long and healthy life. The diversity of strategies within the categories of the process model is furthermore supported by findings showing that they do not form internally consistent factors (see Hennecke et al., 2019; Wenzel et al., 2022). In turn, a more fine-grained analysis of self-regulatory strategies might reveal interesting differences in their efficacy (see Hennecke et al., 2019). Given that most prior research has looked at the efficacy of the broader types of strategies (e.g., situation modification) during conflicts of inhibition (Lopez et al., 2021; Milyavskaya et al., 2021; Williamson & Wilkowski, 2020), we do, however, not know much about the specific types of strategies during the three different types of conflicts. The first aim of the current research is therefore to gather the specific strategies people deploy during all three types of self-control conflicts, that is, self-control conflicts of initiation, persistence, and inhibition.

## Strategy Efficacy and Conflict Type

The second aim of the present studies is to investigate the efficacy of these self-regulatory strategies. Given that some strategies may be used across the three types of self-control conflicts (e.g., reappraisal) we also investigate the extent to which a strategy’s efficacy is moderated by the type of conflict they are used for. Regarding this second aim, we go beyond previous work that has looked at the effects of various strategies but that has, however, usually looked at only one type of self-control conflict (e.g., persistence: Hennecke et al., 2019; inhibition: Milyavskaya, et al., 2021; Williamson & Wilkowski, 2020). A strategy that is helpful in one type of conflict may, however, not be helpful or even be harmful in another (Bonanno & Burton, 2013; Hennecke & Bürgler, 2020). In the work on emotion regulation, for example, it was long purported that problem-focused coping is more beneficial than emotion-focused coping (e.g., Kohn, 1996). More recently, however, it has been shown that if the stressor is uncontrollable, e.g., chronic pain, then emotion-focused coping might be more advisable (Aldridge & Roesch, 2007; Austenfeld & Stanton, 2004; Clarke, 2006). Consequently, emotion regulation research has shifted away from examining the general efficacy of emotion regulation strategies but instead highlighted the importance of a fit between a strategy and a given situation (Aldao et al., 2015; Bonanno & Burton, 2013). In line with this perspective, we assume that the efficacy of any given self-regulatory strategy at any given point in time may also depend on various factors. These may include a person’s trait self-control (Haws et al., 2011), motivational orientation (Scholer & Miele, 2016), goal characteristics (e.g., a goal’s orientation towards approach vs. avoidance, Hennecke, 2019), and many more. Here, we will focus on one feature of self-control conflicts, namely, whether it is a conflict of inhibition, initiation, or persistence.

Why should it matter whether a self-control conflict requires the inhibition of an impulse, the initiation of a disliked activity, or persistence in a disliked activity? Indirect evidence that conflicts of inhibition, initiation, and persistence pose somewhat different challenges comes from research showing that how successfully people self-regulate their behavior across these three types of conflicts is best captured by independent factors (De Ridder et al., 2011; De Boer et al., 2011; Hoyle & Davisson, 2016). In addition, there is also more direct evidence for the notion that the efficacy of strategies can depend on the type of conflict, the strategy is used for (Hennecke et al., 2019; Mischel et al., 1972): The strategy distraction was, for example, shown to be particularly effective in the classic delay-of-gratification paradigm. Children who were instructed to distract themselves by thinking of something else than the marshmallows in front of them, were more patient and more successful in enduring the delay period (Mischel et al., 1972). In the study by Hennecke et al. (2019), however, distraction emerged as the only strategy that was, in fact, negatively related to the participants’ self-regulatory success during conflicts of persistence. So far, however, no study has directly compared the efficacy of distraction or other strategies in the three types of self-control conflict.

While mainly we explore, albeit in a large data set, across strategies whether their effectiveness is moderated by the type of conflict, we also investigate two specific hypotheses in line with these prior findings. First, we hypothesize that the strategy “distraction” is effective when dealing with conflicts of inhibition but even negatively related to self-regulatory success in conflicts of persistence. This should be the case because when inhibition is required, distraction takes attention away from the tempting stimulus. In persistence scenarios, however, distraction takes attention away from the goal-directed activity. Even though this goal-directed activity is, during these self-control conflicts, experienced as aversive, not attending to it might distract the person from staying committed to the current goal pursuit and therefore reduce goal-directed motivation (Hennecke et al., 2019).

Second, we hypothesize that the strategy of “thinking of the positive consequences” is negatively associated with self-regulatory success in conflicts of inhibition but positively associated with self-regulatory success in conflicts of persistence. Both was shown in separate studies: Whereas the strategy was shown to be somewhat detrimental to goal achievement during a low-calorie diet (Freund & Hennecke, 2012), a pursuit that probably involves a lot of inhibition, it was positively associated with self-regulatory success when people reported trying to be persistent in various activities (Hennecke et al., 2019). Aside from these two hypotheses, we chose a data-driven approach to explore the efficacy of all self-regulatory strategies depending on conflict type.

## Creating Strategy-Situation-Fit as an Aspect of Regulatory Flexibility

If it is true that the efficacy of a strategy depends on the conflict it is used for, then self-control should benefit from a fit between a given situation (here: type of conflict) and the strategy that is used to handle it. Accordingly, we hypothesized that individuals who more frequently create strategy-situation-fit by more frequently using strategies for conflicts that they are useful for, should also report higher levels of self-regulatory success overall. This hypothesis is in line with similar notions from emotion regulation, where the ability to create strategy-situation fit has been proposed as a crucial aspect of regulatory flexibility, and in turn, adaptive emotion regulation (Bonanno & Burton, 2013). To the best of our knowledge, evidence for this notion is limited to emotion regulation research (Haines et al., 2016; Troy et al., 2013; Wenzel et al., 2020). Whether being able to create strategy-situation fit is also a predictor of self-control, however, has until now not been investigated.

## The Present Research

In the present research, we investigated the following three research questions: (1) What are the self-regulatory strategies people spontaneously use in their everyday lives, when confronted with different types of self-control conflicts, that is, conflicts of inhibition, initiation, and persistence? (2) Which strategies are most effective in general (i.e., general strategy efficacy)? And does strategy efficacy depend on the type of conflict individuals experience (i.e., strategy efficacy moderated by conflict type)? As described above, we hypothesized that the efficacy of the two strategies “distraction” and “focusing in positive consequences” would be moderated by conflict type. We explored moderator effects for all other types of strategies. And finally, (3) are individuals who flexibly tie the choice of self-regulatory strategies to the types of conflicts they experience more successful in self-control, as hypothesized?

We analyzed three datasets. Our exploratory Pilot Study used the Day Reconstruction Method (DRM, Kahneman et al., 2004), a diary-type method valid for capturing people’s everyday experiences and behaviors, to compile a list of self-regulatory strategies that people report using in their daily lives when being confronted with conflicts of inhibition, initiation, and persistence. In the Main Study, we mega-analyzed two experience sampling datasets (Czikszentmihalyi & Larson, 1987) with very similar sampling schemes and measures to investigate everyday strategy choice, strategy efficacy—in general and as a function of conflict type—and the association between people’s tendency to create situation-strategy fit and their overall self-regulatory success.

## Pilot Study

### Method

This study aimed at generating a list of the strategies that people use in their daily lives to help themselves succeed in self-control conflicts of inhibition, initiation, and persistence. This list of strategies was used as the basis for the Main Study in which participants were asked about their strategy use in daily life.

#### Sample

Based on available funds, we aimed at recruiting 200 participants. Participants were recruited through mailing lists as well as through the student pool at the Department of Psychology at the University of Zurich, Switzerland. Despite our efforts, we were unable to recruit more than *N* = 190 participants (age: *M* = 27.1 years, *SD* = 9.1, 139 female, 50 male, 1 other). These were asked to reconstruct the self-control conflicts they had experienced the day before and to describe the strategies, if any, they had used to help themselves succeed (that is, act in accordance with their goal) during these conflicts. Participants received either course credit (if they were Psychology students) or 40 Swiss Francs as reimbursement.

#### Procedure

After providing their informed consent, participants provided their sociodemographic characteristics. Afterwards, they responded to various questionnaires that are not in the focus of the current research (see OSF for the complete survey, an overview of constructs, and a description of the adaptations made to the original DRM: https://osf.io/dvp2y/). In the part of the study that is central to the current research, we deployed and slightly adapted the DRM and asked participants to describe in some detail, the flow of events and experiences during their previous day (Kahneman et al., 2004). Participants were asked to remember yesterday as a continuous stream of episodes, each of which they should describe in terms of their approximate start and end times and regarding what happened in them. For each episode, we then asked participants to describe what they were doing, where they were, with whom, and whether they had experienced one or more self-conflicts that could either be conflicts of initiation (when they wanted to pull themselves up for the initiation of some activity that they did not feel like doing), of persistence (when they wanted to continue an activity persistently that they did not feel like doing), or of inhibition (when they wanted or had to resist a temptation).

If participants indicated that they had experienced such (a) conflict(s), they were asked to indicate what type it was (initiation, persistence, inhibition, or another type of situation), provide some more information on it and describe up to 10 strategies they used (if any) to help themselves resolve the conflict in the interest of their goal. We asked them (example for initiation type conflict): “Have you deployed any strategies, to help yourself to pull yourself up for the activity that you did not feel like doing? Have you, for example, thought of something special or avoided certain thoughts? Have you attended to or ignored certain aspects of the situation? Have you changed how you feel? Or what you do or the way you do it?” We designed these instructions to cover the different categories of strategies proposed in Duckworth et al. (2016) process model of self-regulation. Therefore, the question prompted participants to think of strategies that would modify the situation or their own behavior (“what you do or the way you do it”), that would modulate their attention (“attended to or ignored certain aspects of the situation”), and that would change the way they think (“thought of something special or avoided certain thoughts”). In addition, given that participants in Hennecke et al. (2019) often referred to emotion regulation strategies with no clear indication of their locus within the process model, we added a prompt to also consider such strategies (“how you feel”).

#### Coding of Responses

Four coders were involved in coding the strategies reported by participants. In an initial round of coding, two of the authors independently tried to assign each strategy from a list of 150 randomly chosen strategies (50 for each conflict type) to a higher order strategy type description. If possible, this was done by assigning strategies to strategy types from Hennecke et al. (2019). After this step, these two coders discussed inconsistencies or disagreements regarding strategy assignments and new strategy type descriptions. After having agreed on a list of strategy types, two other coders (both with undergraduate degrees with a major in Psychology) independently coded the same list of strategies. Given that interrater agreement after this first round was unsatisfactory (Kappas ranging from .48 to .62), the four coders discussed potential causes of disagreement and refined the coding scheme. In the next round, two coders coded another list of 150 randomly chosen strategies (50 for each type). Interrater agreement was still unsatisfactory (Kappa = .47). Again, the four coders discussed potential causes of disagreements and refined the coding scheme. Eventually, in a last round of coding for 150 strategies, two independent coders attained an interrater agreement of Kappa = .60, a score that is considered “fair to good agreement beyond chance” (Fleiss et al., 1981, p. 604). Note that our goal was not to develop a failproof coding system but rather to extract a comprehensive list of the kinds of strategies people use in their everyday life that are sufficiently distinct from each other. We therefore did not have to reconcile any disagreements between coders, as long as each of two disagreeing coders was able to find a strategy type that, in their perspective, suited the strategy. By advancing our coding system, we made sure that for each strategy, that did not seem like a nonsense response, there was a category to which it could be assigned.

### Results and Brief Discussion

In sum, participants reported 508 self-control conflicts. 210 (41.3%) of these were categorized as initiation conflicts, 129 (25.4%) as inhibition conflicts, and 105 (20.7%) as persistence conflicts. For 64 (12.6%) conflicts, participants indicated that they were neither conflicts of initiation, persistence, or inhibition but a different type of situation.^1^ Across conflicts, participants described 525 different strategies, with an average of 3.8 strategies (*SD* = 3.5) per conflict. 256 (48.8%) of these strategies referred to conflicts of initiation, 127 (24.2%) to conflicts of persistence, and 92 (17.5%) to conflicts of inhibition. Through the various iterations described above, the coding schema arrived at a list of 26 different strategies, most of which could be used for all three types of conflicts. These were: “Changing the environment,” “removing or reducing distractions or temptations,” “seeking social support,” “adopting a process focus,” “distracting oneself from the activity or temptation,” “anticipating self-reward,” “focusing on negative consequences,” “focusing on positive consequences,” “goal setting,” “monitoring one’s goal progress,” “planning/scheduling,” “putting oneself under (social, time, and performance) pressure,” “reasoning in favor of the target activity/against the temptation,” “reminding oneself of a commitment to oneself or others,” “self-affirmation,” “self-talk,” “thinking of the near finish,” and “suppressing the impulse to quit or to give in to the temptation/forcing oneself to initiate the aversive activity.” The strategies “changing the activity itself” and “task enrichment” were suitable only for conflicts of initiation and persistence. In addition, the strategies “substituting the temptation,” “indulging a little,” and “taking a break” were suitable for only one type of conflict and, in turn, not considered in the main study. The full list of strategies, and their descriptions from the coding scheme is displayed in Table 1S on the OSF.

The study shows that there is great variety in the ways that people strategically self-regulate during conflicts of inhibition, initiation, and persistence. In our perspective, the self-regulatory strategies are located on a useful level of abstraction, that was prompted by participants’ own responses and that seems to provide a good balance between parsimony and differentiation (see also Hennecke et al., 2019). It should therefore be useful for application in an experience sampling study where participants are asked to choose from a list those strategies they deployed in a recent self-control conflict.

Note that even though we applied a bottom-up approach, the strategies reported furthermore have their equivalents in the self-regulation literature. Not only was it possible to assign the strategies to the categories or stages proposed in the process model of self-regulation (Duckworth et al., 2016) (except for “emotion regulation” which can occur at various stages and in a variety of ways and was, due to a lack of specificity, not considered in the Main Study), but many of the more specific strategies, for example, “changing the environment”, “reducing distractions”, “distraction”, “anticipating self-reward”, or “goal setting”, have already been reported and studied elsewhere (e.g., Bandura, 1976; Ent et al., 2015; Goldfried & Merbaum, 1973; Grunschel et al., 2016; Klein et al., 1999; Kuhl, 1984; Locke & Latham, 2002; Mischel et al., 1972; Pintrich, 2000; Schunk, 1990; Zimmerman, 1990, 2000).

## Main Study

The Main Study aimed at answering our two research questions regarding strategy choice and strategy efficacy, using the strategies derived in the Pilot Study. To answer these research questions, we conducted two experience sampling studies. Participants in these studies were asked multiple times a day, for multiple days in a row, whether they had just experienced a self-control conflict and if, so, whether it was a conflict of initiation, persistence, or inhibition. They furthermore reported which (if any) strategies they had used to deal with this self-control conflict and on their self-regulatory success. We were interested in which strategy was selected in response to the conflict type and whether the efficacy of a strategy was moderated by the type of self-control conflict for which it was deployed. In addition to an exploratory approach, we tested the hypotheses that the efficacy of the two strategies “distraction” and “focusing in positive consequences” would be moderated by conflict type. Finally, we tested whether creating strategy-situation-fit was beneficial for participants’ self-regulatory success.

### Method

#### Participants

A-priori power analysis for studies with repeated measures is difficult when within-person correlations of these repeated measures are unknown. We therefore based our target sample size on previous research with similar research questions and study designs (Friese & Hofmann, 2016; Hennecke et al., 2019; Hofmann et al., 2012; Milyavskaya et al., 2015) and available resources. In this research, sample sizes ranged from *N* = 101 to *N* = 297 participants. Based on available resources, we were able to aim at samples of *N* = 250 in Study 1 and *N* = 500 in Study 2. We recruited participants in lectures and seminars at the University of Siegen, official student mailing lists, as well as the university’s Facebook and Twitter accounts. To recruit non-students, we furthermore placed advertisements in local newspapers and a local radio station. In order to be eligible for study participation, participants had to own an Android smartphone (the application used for experience sampling is only available on Android-operated systems) and be at least 18 years old. In both studies, participants could either receive course credit or for financial reimbursement (up to €50, based on the number of daily questionnaires the participant filled out during the study).

##### Dataset 1

We ceased our recruitment efforts when *N* = 261 participants had signed up for the study. Out of these, *N* = 224 completed the baseline questionnaire and provided at least one conflict and were, thus, included in the analyses (age: *M* = 26.0 years, *SD* = 7.7 years, age range: 18–62 years, 143 female). This dataset was already used in Bürgler et al. (2021), for a different research question.

##### Dataset 2

We ceased our recruitment efforts when *N* = 503 participants had signed up for the study and there were no additional enrollments. Out of these, *N* = 492 completed the baseline questionnaire and provided at least one conflict and were, thus, included in the analyses (age: *M* = 24.8 years, *SD* = 6.4 years, age range: 18–64 years, 339 female).

#### Procedure

Participants could sign up for the study by providing their informed consent on a university website. Afterwards, participants received a personalized link to the baseline survey, which was presented via LimeSurvey (2003). Once they had finished the baseline survey, participants received per e-mail a QR code which they were asked to scan with the Movisens application. Participants were able to choose a preferred 14-hour time window during which they would receive their questionnaires (between 7 a.m. and 9 p.m.: 15%; between 8 a.m. and 10 p.m.: 29%; between 9 a.m. and 11 p.m.: 56%). From then on, the application sent participants eight questionnaires per day over the course of 10 days by informing them with a push message. These questionnaires were sent at random points in time, with the only constraint that there had to be at least 1 hour in between two questionnaires. Participants could accept, dismiss, or delay the questionnaire for up to 10 minutes. After 10 minutes, the questionnaire was no longer available.

If participants filled in less than 60 out of the possible 80 questionnaires during the 10-day period, an 11^th^ day of data collection was added. This was the case for 51% of participants. When participants had finished the experience sampling part, they received one last thank-you email that also informed them about when they could expect to be reimbursed.

**Dataset 2.** The procedure of Dataset 2 was the same as for Dataset 1, with some minor differences: First, we choose not to add an 11^th^ day to the assessment of the daily questionnaires for any of the participants because adding an 11^th^ led to complications with the Movisens app with some smartphone models (some participants did not receive all questionnaires for that 11^th^ day). Furthermore, we assumed that the increased number of participants would make up for the lower number of observations for each participant. Second, a very brief questionnaire in the evening and a follow-up questionnaire, both of which were not relevant to the current research questions, were added. Third, because of additional research questions for which the assessment of additional items was necessary, but we did not want to overburden participants (Eisele et al., 2022), we split the questionnaire into two forms that were randomly presented to participants (see SOM). Fourth, participants were able to choose from four instead of three different time windows, either between 7 a.m. and 9 p.m.: 14%, 8 a.m. and 10 p.m.: 28%, 9 a.m. and 11 p.m.: 28%, or 10 a.m. and 12 p.m.: 30%. Fifth, participants were able to delay the questionnaire by up to 15 minutes instead of 10 minutes, giving participants more flexibility on when to answer the questionnaires in the hopes of increasing response rates.

#### Measures

##### Type of self-control conflicts experienced

The first question in each experience sampling questionnaire asked participants whether they had experienced, within the last hour, a self-control conflict. We described it as an “inner conflict between what you would like to do and what you should be doing instead.” Participants then indicated whether they a) had the feeling they “should pull themselves up or pulled themselves up for an activity that they did not feel like doing (e.g., because it was unpleasant, boring, effortful, or frustrating)” (initiation conflict), b) had the feeling they “should continue with an activity or continued an activity that they did not feel like doing (e.g., because it was unpleasant, boring, effortful, or frustrating)” (persistence conflict), c) had “experienced a temptation or resisted one” (inhibition conflict), or d) did not experience any of these types of conflicts.

If participants indicated not having experienced any of the conflict types, they received a couple of filler questions about the activity they engaged in right before the signal (type of activity, goals of the activity, approach, and avoidance orientation). We included these to reduce the incentive for falsely responding that one did not experience any self-control conflict to reduce one’s own burden of responding.

##### Further description of conflict

If participants indicated having experienced one of the three conflict types, depending on the type of conflict reported, participants were asked to give additional details on the activity they wanted to initiate or persist in or on the temptation they had experienced. Participants were asked to categorize the activity they wanted to initiate or persist in by choosing one or more types of activities from a list including, for example, “commuting,” “work/job,” “studying,” “personal hygiene,” and others (Hennecke et al., 2019; Kahneman et al., 2004). Alternatively, participants were asked to categorize the temptation they had resisted or wanted to resist by choosing one or more types of temptations from a list including, for example, “food,” “coffee,” “alcohol,” “drugs,” “media use,” “sex,” and others (Hofmann et al., 2012). Additionally, all participants were asked to indicate what type of goal the activity or resisting the temptation aimed at by choosing goal categories from another list, containing, for example, “relationship with partner,” “social (not with partner),” “academic/work-related,” “health/fitness,” “financial,” “enjoyment/pleasure,” and others (W. Hofmann, personal communication, July 14, 2017).

##### Self-regulatory strategies

If participants indicated having experienced a self-control conflict, we asked them, depending on the type of conflict, to select from a list which strategies, if any, they had deployed to do so. These strategies were identical to the strategies derived in Study 1 (see Table 1S). Participants could select multiple strategies from a list for each conflict they had experienced. The descriptions of the strategies that participants could choose from are displayed in Table 1S on the OSF. They choose *M* = 2.8 (*SD* = 2.1) strategies per self-control conflict.

##### Subjective self-regulatory success

Subjective self-regulatory success in resolving the self-control conflicts was assessed with two items, whose exact wording depended of the type of conflict, namely, “How well did you manage to initiate the aversive activity/to persist in the aversive activity/to resist the temptation?” (1 = not well at all, 7 = very well) and “How satisfied are you with how well you managed to initiate the aversive activity/to persist in the aversive activity/to resist the temptation?” (1 = not satisfied at all, 7 = very satisfied). Items regarding *subjective success* and *satisfaction* were correlated with *r*_*SB*_ = .88 (within-person; Spearman–Brown corrected) and *r*_*SB*_ = .93 (between-person) for conflicts of initiation, *r*_*SB*_ = .86 and *r*_*SB*_ = .92 for conflicts of persistence and with *r*_*SB*_ = .81 and *r*_*SB*_ = .84 for conflicts of inhibition, and, in turn, aggregated into one score.

##### Statistical analyses

Because observations were nested within individuals, we conducted multilevel regression analyses to account for the hierarchical nature of the data. Given that both datasets contained the same measures and followed the same study protocol, we opted to perform a *mega-analysis* or *integrative data analysis* (Curran & Hussong, 2009). Such an approach is recommended when the data are available given that it leads to lower standard error estimates compared to a meta-analysis and, thus, increased power (Boedhoe et al., 2019). In addition, this approach increased the observations per cell, which were sometimes low for some conflict type × strategy interactions. For example, “anticipating self-reward” was reported 26 times for inhibition conflicts in the first, and 17 times in the second dataset. Thus, the data from both datasets were appended during data preparation and the following models were run on the combined dataset. To provide a measure of heterogeneity of our tests, we included dataset (1 = Dataset 1, 2 = Dataset 2) as a predictor and included its interaction with the predictors of interest. We present the mean associations in the results section and the associations based on the individual datasets on OSF (https://osf.io/dvp2y/). For all analyses, we used a significance level of .05. Given the exploratory nature of the analyses, we controlled for Type I error inflation by using two methods. First, when comparing the associations between the 22 self-regulatory strategies, we used the procedure by Benjamini and Hochberg (1995), which corrects the inflation by controlling the false discovery rate (FDR). Second, when comparing the interactions between conflict type and a particular strategy, we used the Šidák correction (Šidák, 1967) to adjust for the three possible comparisons. Finally, given that strategy efficacy might depend on whether only a single strategy was used or whether other strategies were selected, we included such a factor (0 = none of the strategies were selected, 1 = only the strategy of interest was used, 2 = at least one other self-regulatory strategy was used in addition) as a control variable.

##### Variance component analysis

Among other factors, variance in strategy use can be explained by differences between individuals and conflict type as well as between their interaction. To quantify how much variance can be attributed to these factors, we conducted variance components analyses (Searle et al., 2009) by computing multilevel models in which conflict type was nested within participants as well as was crossed with participants, whereas observations were nested within conflict type (Marchenko, 2006). Consequently, the random intercept variance of participants in this model estimates the variance that can be attributed to differences between individuals. The random intercept variance of conflict type that is crossed with participants estimates the variance that can be attributed to differences between conflict types. Finally, the random intercept variance of conflict type that is nested within participants estimates the variance that can be attributed to person × conflict type interactions, which could reflect, among other things, different strategy-conflict type relations. Note that strategies which could only be used in one type of conflict (e.g., “indulging a little”) were not considered in the analyses. Regarding the variance in subjective self-regulatory success, we also examined how much of its total variance could be explained by differences in self-regulatory strategies as well as by person × conflict type × strategy interactions.

##### General strategy efficacy

To capture the general subjective efficacy of a self-regulatory strategy across all conflict types, we computed three-level models in Stata 17 (Stata Corporation, College Station, TX), where observations were nested conflict type which were nested within individuals. Using the *mixed* command in Stata 17 and the maximum likelihood option, we predicted self-regulatory success by self-regulatory strategy use (0 = strategy was not used, 1 = strategy was used). In this model, the slope was allowed to vary randomly between individuals. In addition, we included the dataset factor and its interaction with self-regulatory strategy use as well as the multiple strategy use factor as control variables. To control for Type I error inflation, we used the FDR.

##### Strategy efficacy and conflict type

Here, we computed three-level models (observations nested within conflict types nested within individuals), where subjective self-regulatory success was predicted by the two-way interaction between a given self-regulatory strategy and the dummy-coded conflict type (with initiation conflicts as the baseline) in the first step, allowing for a random slope. Given our hypotheses and the popularity of inhibition in self-control research, we chose inhibition as the base category. Both the self-regulatory strategy and conflict type were allowed to vary randomly between individuals. We then computed an omnibus test of the two-way interaction, followed by an estimation of the simple slopes of the relationship between strategy and subjective success for each conflict type. The two-way interactions were FDR-controlled (22 comparisons in total), whereas the simple main effects of conflict type for each strategy was Šidák-controlled (3 comparisons per strategy).

##### Strategy choice and conflict type

Next, we examined how likely strategies were implemented and how strategy choice was moderated by conflict type. To that end, we computed three-level logistic regression models (observations nested within conflict types nested within individuals), where strategy use was predicted by dummy-coded conflict type. Then, to estimate the likelihood of endorsing a particular strategy, we estimated the predicted probabilities by using the margins command in Stata 17 and subtracted the average probability across all strategies to test whether a particular strategy was endorsed more often compared to the average. Finally, we estimated the omnibus test for conflict type and the predicted probabilities for each conflict type. Again, strategy choice and the two-way interactions were FDR-controlled and the simple main effects were Šidák-controlled.

##### Effects of regulatory flexibility through strategy-situation fit on subjective self-regulatory success

To examine whether people benefit from creating strategy-situation fit, we computed multilevel dynamic structural equation models (DSEM) in Mplus Version 8.7 (Muthén & Muthén, 2017), which allows the simultaneous prediction of multiple outcomes in multilevel time series (Hamaker et al., 2018). In our case, we predicted strategy use by the dummy-coded conflict type. In the second step, we used the person-specific association between strategy choice and conflict type as predictors of subjective self-regulatory success on the between-person level, which also included the respective strategy and the dummy-coded initiation and persistence variable as well as the dataset factor and its interactions to control for dataset differences. This way we can answer the question whether individuals who use a strategy more often in situations where this strategy is particularly helpful, for example more often for conflicts of initiation than for conflicts of inhibition, report greater self-regulatory success on average above and beyond the mean use of that strategy and conflict type.

Another advantage of simultaneously predicting multiple outcomes in one model in Mplus is that the person-specific slope in the first step is not treated as a perfectly reliably assessed observed variable but instead as a variable that is affected by measurement error due to the possibly different number of observations for each participant the measure is based on. Recent research has shown that neglecting this information leads to inflated Type-I errors and underestimated standard errors (Liu et al., 2021), thereby avoiding biased estimates of person-level constructs (Lüdtke et al., 2008).

For the estimation in Mplus, we used a Bayesian estimator, a Gibbs sampler with two MCMC chains, as well as the default priors so that the results were driven by the data. Furthermore, we used a Potential Scale Reduction (PSR) of 1.005 by setting the BCONVERGENCE option in Mplus to 0.0025 as the convergence criterion. This criterion is lower than the default PSR of 1.1 in Mplus. However, we chose the lower criterion because prior research has shown that lower values than 1.1 lead to greater precision (Zitzmann & Hecht, 2019). However, values lower than 1.005 do not sufficiently improve precision (Zitzmann & Hecht, 2019); thus, the chosen criterion provides the best tradeoff between precision and computational power. Moreover, we discarded every 50^th^ iteration of the estimation (i.e., by using a thinning of 50) to reduce autocorrelation between the iterations. We, then, repeated the model with a total of 4000 fixed iterations to check that the PSR value did not increase. This was not the case and the model fit based on statistical criterions (PSR below 1.005 and quickly decaying lags below *r* < .20, which was the case) as well as on visual inspection of the trace plots (which looked fine by resembling a fat, hairy caterpillar) did not show any sign of misspecification or poor model fit. Thus, we report the estimates along with their 95% credible intervals (95% CI) and the *p* values of the repeated models, which were FDR-controlled (5 comparisons). The model outputs and graphs as well as the analysis scripts and the data can be found on the OSF project page (https://osf.io/dvp2y/).

## Results

### Descriptive Statistics

Participants provided a total of 41,461 reports. They experienced a self-control conflict at 33.9% (*n* = 14,067) of measurements, with a mean number of conflicts per participant of *M* = 19.6. Conflicts of initiation (*n* = 7,310, 17.6%) were the most common type of self-control conflict, followed by conflicts of persistence (*n* = 3,832, 9.2%) and conflicts of inhibition (*n* = 2,925, 7.1%). Initiation conflicts were reported most frequently for studying (34.1%), commuting (11.6%), and work (11.0%). Persistence conflicts were reported most frequently during studying (50.8%), work (13.5%), and classes (10.7%). Temptations tended to occur primarily in the domains of food (43.3%), media (13.8%), and sleep (11.4%).

Initiation conflicts were reported most frequently during the pursuit of academic goals (53.9%), housework goals (14.9%), and health goals (14.5%). Persistence conflicts were reported most frequently during the pursuit of academic goals (73.0%), financial goals (9.9%), and social goals (9.8%). Inhibition conflicts were reported most frequently during the pursuit of health goals (54.5%), academic goals (24.9%), and social goals (8.1%).

#### Variance Component Analyses

Regarding variance in subjective self-regulatory success, 47.1% of the total variance in subjective self-regulatory success could be explained by differences between individuals, conflict type, and self-regulatory strategies. Person differences explained 11.6% of this variance, whereas conflict type and self-regulatory strategy differences only explained very limited variance in subjective self-regulatory success, with 0.2% and 0.03%, respectively. Importantly, person × type interactions explained the most variance in subjective self-regulatory success with 34.3%, which highlights the importance to examine the different ways individuals respond to the three conflict types in daily life. However, person × type × strategy interactions only explained an additional 1.0% of the total variance in subjective self-regulatory success. The results were consistent across datasets, with person × type interactions and then person differences explaining the most variance in subjective self-regulatory success.

**Figure 1.**
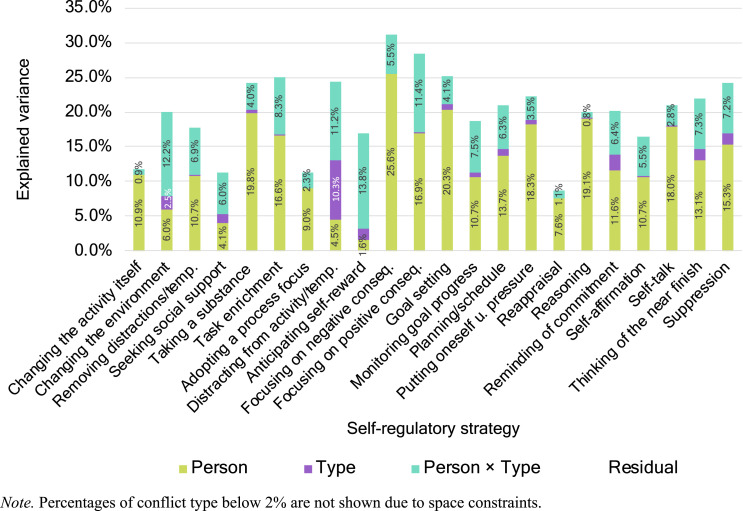
Variance components (%) of person, conflict type, and person × conflict type differences for each self-regulatory strategy. *Note*. Percentages of conflict type below 2% are not shown due to space constraints.

Regarding variance in the selection of self-regulatory strategies, Figure 1 indicates that, on average, person differences contributed most strongly to explaining strategy choice differences, followed by person × type interactions and by conflict type differences, with the, again, only explaining limited variances. However, as Figure 1 demonstrates, there was considerable variability, depending on the strategy, regarding the total level of explained variance as well as to the specific make-up. For example, whereas 31.1% of the total variance in the strategy “focusing on negative consequences” could be explained, only 8.7% of the total variance in “reappraisal” could be explained by differences between persons, types of conflict, and their interaction. Person differences accounted for between 1.6% (for “anticipating self-reward”) and 25.6% (for “focusing on negative consequences”) of the variance in the respective strategy. Differences in conflict type accounted for between 0.0% and 10.3% of variance in the respective self-regulatory strategy. Lastly, person × conflict type interactions accounted for between 0.8% (for “reasoning”) and 13.8% (for “anticipating self-reward”) of the variance. Together, the data show that considering all three sources of variance is helpful for understanding differences in strategy choice within and across the three types of self-control conflicts, with conflict type adding, on average, 1.0% and person × type interactions adding, on average, 6.1% explained variance in the respective strategy above person differences, which explained 12.9% on average.

#### Strategy Efficacy

##### Main effects

**Figure 2.**
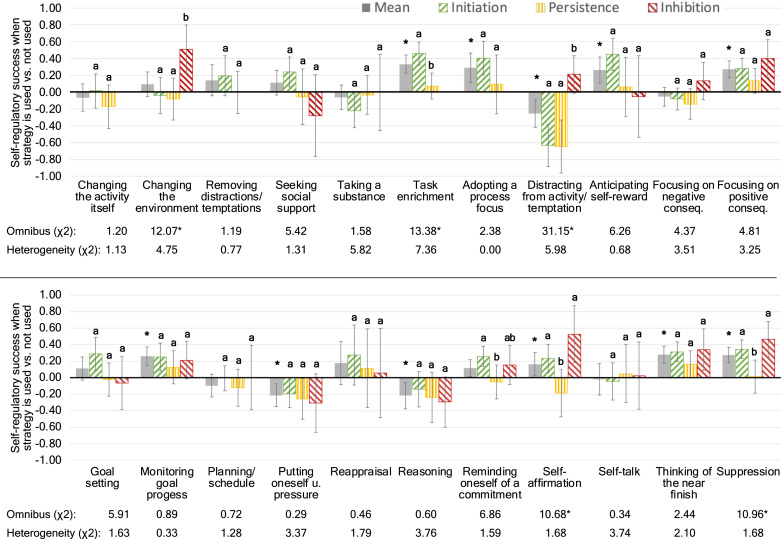
Coefficient plot showing the regression coefficient (the dots) and its 95% CI (the whiskers) of the two-way interaction between a self-regulatory strategy and conflict type in the prediction of subjective self-regulatory success on the within-person level. *Note*. tempt. = temptation. Whiskers represent 95% confidence intervals. Bars with different letters differ significantly from each other after Šidák correction. * Significant after Benjamini-Hochberg correction (Omnibus and heterogeneity test as well as mean efficacy).

Figure 2 shows the associations between strategies and subjective self-regulatory success on the within-person level. Based on participants’ self-reports, eight of the 22 strategies were generally adaptive, with “suppression,” “task enrichment,” “focusing on positive consequences,” and “thinking of the near finish” being the most adaptive strategies. Three strategies, “distracting oneself from the activity/temptation,” “putting oneself under pressure,” and “reasoning in favor of target activity or against the temptation” were maladaptive, that is, endorsing these strategies was significantly associated with lower subjective self-regulatory success than not endorsing them. The remaining strategies had no effects across conflict types. For example, endorsing strategies like “changing the activity” or “planning” more strongly than an individual is typically doing was not significantly associated with increased subjective self-regulatory success. Importantly, none of the two-way interactions with the dataset factor indicated significant heterogeneity (not shown in Figure 2), except for “suppression,” which was, according to self-reports, significantly more successfully implemented by participants in Dataset 1, *b* = 0.37, *SE* = 0.07, *p* < .001, 95% CI [0.24, 0.50], than in Dataset 2, *b* = 0.15, *SE* = 0.06, *p* = .023, 95% CI [0.02, 0.27]. However, suppression was significantly associated with subjective self-regulatory success in both datasets.

##### Moderation: Conflict Type

Next, we examined whether subjective strategy efficacy was moderated by conflict type. The results in Figure 2 show that the efficacy of most self-regulatory strategies did not differ between the conflict types. However, the subjective efficacy of five strategies was significantly moderated by conflict type.

“Changing the environment” was only positively and significantly associated with successfully regulating conflicts of inhibition, *b* = 0.51, *SE* = 0.14, *p*_*Šidák*_ = .001, 95% CI_Šidák_ [0.17, 0.86], but not of initiation, *b* = −0.04, *SE* = 0.11, *p*_*Šidák*_ = .978, 95% CI_Šidák_ [−0.30, 0.22], or persistence, *β* = −0.08, *SE* = 0.13, *p*_*Šidák*_ = .889, 95% CI_Šidák_ [−0.38, 0.22]. Thus, participants reported being significantly more successful when changing their environment while confronted with conflicts of inhibition compared to initiation, *b* = 0.55, *SE* = 0.18, *p*_*Šidák*_ = .007, 95% CI_Šidák_ [0.12, 0.98], or persistence, *b* = 0.59, *SE* = 0.19, *p*_*Šidák*_ = .006, 95% CI_Šidák_ [0.14, 1.05], with no difference between the latter, *p*_*Šidák*_ = .992. In other words: When trying to inhibit an unwanted impulse, people presumably benefitted from moving to a different location. When trying to initiate or persist a disliked activity, moving to a different environment did not help.

“Distracting oneself from the activity or temptation” was negatively and significantly associated with subjective self-regulatory success during conflicts of initiation, *b* = −0.63, SE = 0.13, *p*_*Šidák*_ < .001, 95% CI_Šidák_ [−0.94, −0.33], and of persistence, *b* = −0.65, *SE* = 0.16, *p*_*Šidák*_ < .001, 95% CI [−1.03, −0.26]. However, it was positively but not significantly associated with subjective self-regulatory success during conflicts of inhibition, *b* = 0.21, *SE* = 0.11, *p*_*Šidák*_ = .175, 95% CI_Šidák_ [−0.06, 0.48]. Thus, distraction was both maladaptive (initiation and persistence conflicts) and adaptive (inhibition conflicts), depending on the type of conflict. This partially supports our hypothesis that distraction should be a positive predictor of subjective self-regulatory success for conflicts of inhibition but not for conflicts of persistence, where we predicted it would be a negative predictor.

Next, as indicated in Figure 2, “task enrichment” was only significantly effective for conflicts of initiation, *b* = 0.46, *SE* = 0.07, *p*_*Šidák*_ < .001, 95% CI_Šidák_ [0.30, 0.61], but not of persistence, *b* = 0.07, *SE* = 0.08, *p*_*Šidák*_ = .585, 95% CI_Šidák_ [−0.10, 0.25]. It was not investigated for conflicts of inhibition, where there is no task to be enriched. Moreover, “self-affirmation” was only significantly effective when dealing with conflicts of initiation, *b* = 0.23, *SE* = 0.09, *p*_*Šidák*_ = .023, 95% CI_Šidák_ [0.02, 0.44], and inhibition, *b* = 0.52, *SE* = 0.18, *p*_*Šidák*_ = .009, 95% CI_Šidák_ [0.10, 0.94], but not of persistence, *b* = −0.19, *SE* = 0.15, *p*_*Šidák*_ = .482, 95% CI_Šidák_ [−0.54, 0.16]. Finally, “suppressing the impulse to quit or to give in to the temptation/forcing oneself to initiate the aversive activity” showed the same pattern as “self-affirmation,” such that it was only subjectively effective during conflicts of initiation and inhibition but not during conflicts of persistence. Given that we did not find that the efficacy of “focusing on the positive consequences” was moderated by conflict type (see Figure 2), the hypothesis that this strategy is subjectively adaptive for conflicts of persistence (as found in Hennecke et al., 2019) but not for conflicts of inhibition (as suggested by Freund & Hennecke, 2012) was not supported.

Importantly, none of the three-way interactions between conflict type, self-regulatory strategy, and the dataset factor was significant (Figure 2) and, hence, the two-way interactions between conflict type and self-regulatory strategy did not differ significantly between the two datasets.

#### Strategy Choice

##### Main effects

**Figure 3.**
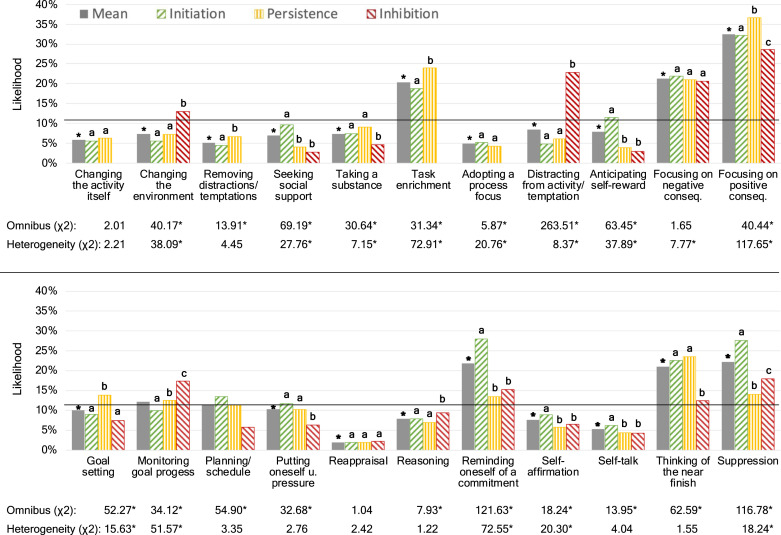
Bar graphs showing the predicted likelihood of using a self-regulatory strategy as a function of conflict type. *Note*. Bars with different letters differ significantly from each other after Šidák correction. * Significant after Benjamini-Hochberg correction (omnibus and heterogeneity test as well as mean efficacy) or after Šidák correction (conflict type differences within a given self-regulatory strategy).

Next, we wanted to examine how often strategies were selected and whether strategies that were experienced as generally more effective were also selected more often. Figure 3 and Table 1 show a large variability in strategy selection: Whereas “reappraisal” was selected in only 2.0% of conflicts participants attempted to regulate, “focusing on positive consequences” was selected in almost a third of those instances.

**Table 1.** Summary of Main Results in the Main Study.

Strategy	Stage	Choice, %	Freq. Rank	Efficacy	Efficacy Rank	Efficacy: Type Moderation	Choice: Type Moderation	Flexibility
Focusing on positive consequences	Cognitive	32.4	1	0.27	5	No	Yes	
Suppression	Response	22.2	2	0.27	4	Yes	Yes	No
Reminding oneself of a commitment to oneself or to others	Cognitive	21.9	3	0.11	11	No	Yes	
Focusing on negative consequences	Cognitive	21.4	4	−0.05	16	No	No	
Thinking of the near finish	Cognitive	21.0	5	0.28	3	No	Yes	
Task enrichment	Situation	20.3	6	0.33	1	Yes	Yes	No
Monitoring one’s goal progress	Cognitive	12.2	7	0.26	7	No	Yes	
Planning/scheduling	Cognitive	11.4	8	−0.10	19	No	Yes	
Putting oneself under pressure	Cognitive	10.3	9	−0.21	20	No	Yes	
Goal setting	Cognitive	10.0	10	0.11	13	No	Yes	
Distracting oneself from the activity or temptation	Attention	8.5	11	−0.25	22	Yes	Yes	Yes
Anticipating self-reward	Cognitive	7.9	12	0.26	6	No	Yes	
Reasoning in favor of target activity or against the temptation	Cognitive	7.9	13	−0.22	21	No	Yes	
Self-affirmation	Cognitive	7.6	14	0.16	9	Yes	Yes	No
Taking a substance	Situation	7.3	15	−0.06	17	No	Yes	
Changing the environment	Situation	7.3	16	0.09	14	Yes	Yes	Yes
Seeking social support	Situation	7.0	17	0.11	12	No	Yes	
Changing the activity itself	Situation	5.8	18	−0.07	18	No	No	
Self-talk	Cognitive	5.3	19	−0.02	15	No	Yes	
Removing or reducing distractions or temptations	Situation	5.2	20	0.14	10	No	Yes	
Adopting a process focus	Attention	5.0	21	0.29	2	No	Yes	
Reappraisal	Cognitive	2.0	22	0.18	8	No	No	

*Note.* Freq = frequency; situation = situation selection or modification strategies; attention = attentional deployment strategies; cognitive = cognitive change strategies; response = response modulation; suppression = suppressing the impulse to quit or to give on to the temptation/forcing oneself to initiate the aversive activity.

Regarding general strategy efficacy, we found that “focusing on positive consequences,” “suppression,” “thinking of the near finish,” and “task enrichment” were, according to participants’ reports, the four most adaptive strategies in our sample. Table 1 shows that these four strategies were not only experienced as highly effective but that they were also highly popular, in that they were selected most, second most, fifth most often, and sixth most often, respectively. To test the hypothesis that strategies that were experienced as more effective were also selected more often, we computed a meta regression where we predicted the general likelihood of selecting a particular self-regulatory strategy (mean in Figure 3, i.e., the values in the choice and efficacy columns in Table 1, respectively) by the general subjective efficacy coefficient of each strategy (mean in Figure 2). This yielded a positive and significant coefficient, *b* = 0.17, *SE* = 0.07, *p* = .018, 95% CI [0.03, 0.30], *r* = .39, indicating that strategies that were perceived as more effective were selected more likely by the participants (and vice versa).

##### Moderation: Conflict Type

Figure 3 also shows how the selection of self-regulatory strategies was moderated by conflict type. Here, we focus on the five self-regulatory strategies, whose subjective efficacy was significantly moderated by conflict type. The full results can be found in the online on the OSF project page.

Regarding “changing the environment,” we found that this strategy was only perceived as effective in regulating inhibition conflicts (Figure 2). Figure 3 shows that this pattern is reflected in participants’ choice as well, as participants were more likely to select this strategy when confronted with inhibition than with initiation or persistence conflicts.

Similarly, “distracting oneself from the activity or temptation” was not only perceived as more effective during conflicts of inhibition compared to conflicts of initiation or persistence, but participants also selected it more often, as evidenced in Figure 3. In a similar vein, “suppression” was significantly less selected for conflicts of persistence than for other conflicts (Figure 3), which was also the only conflict type that “suppression” was not effective for in regulating them (Figure 2).

Regarding “self-affirmation,” we found that it was subjectively more effective for regulating conflicts of initiation and inhibition but not of persistence. However, Figure 3 shows that participants were only more likely choose this strategy, on average, when they were confronted with conflicts of initiation but not of inhibition compared to persistence conflicts. A similar disconnect was found for “task enrichment,” which was selected more often for conflicts of persistence compared to initiation (Figure 3), although Figure 2 shows that it was only effective for conflicts of initiation but not persistence.

Taken together, we found partial evidence for the notion that participants selectively choose strategies for the types of conflicts they also perceive them to be more effective for, particularly for initiation conflicts but to a lesser extent for inhibition conflicts. However, as Figure 3 indicates, these results were often heterogenous, indicating the presence of moderators.

##### Strategy-Situation Fit and Regulatory Flexibility

Finally, we examined whether individuals who favored strategies that were reported to be more effective for specific conflict types reported greater self-regulatory success in general, constituting our operationalization of strategy-situation fit or self-regulatory flexibility. We focused on the five strategies where we found conflict type differences in their efficacy. Given that Mplus does not have an option for applying error correction, we set the alpha = .025 for the following analyses given that there were two comparisons of interest per strategy.

For “changing the environment,” we found evidence for strategy-situation fit: Not only was this strategy experienced as more effective in regulating inhibition conflicts, but participants who selected this strategy more often when confronted with inhibition conflicts reported higher self-regulatory success on average than participants who chose it less often, but only compared to conflicts of persistence, *b* = 2.10, *SE* = 1.26, *p* = .021, 95% CI [0.09, 5.04], but not of initiation, *b* = 1.07, *SE* = 1.17, *p* = .144, 95% CI [−0.92, 3.75]. A similar pattern was found for “distracting from the activity or temptation.” Participants who selected this strategy more likely when confronted with conflicts of inhibition also reported higher subjective self-regulatory success compared to when confronted with conflicts of initiation, *b* = 1.52, *SE* = 0.84, *p* = .022, 95% CI [0.05, 3.32] or of persistence, *b* = 1.69, *SE* = 1.03, *p* = .029, 95% CI [−0.07, 4.09], although the latter coefficient was not significant when applying Šidák correction. These results demonstrate the importance of strategy-situation fit of these two strategies.

However, we did not find evidence for strategy-situation fit for the other three strategies “task enrichment,” “self-affirmation,” and “suppression,” as the person-specific slopes of the relationship between strategy use and conflict type were not significantly associated with subjective self-regulatory success on the between-person level. Although “task enrichment” was more effective for conflicts of initiation than of persistence, participants who selected “task enrichment” more likely when confronted with conflicts of initiation compared to persistence did not report significantly higher levels of subjective self-regulatory success, *b* = 0.81, *SE* = 0.52, *p* = .055, 95% CI [−0.20, 1.83]. Moreover, although “self-affirmation” was less effective for persistence conflicts but selecting it more likely in these situations was not significantly associated with higher self-regulatory success in general, *b* = 1.04, *SE* = 1.48, *p* = .220, 95% CI [−1.72, 4.16] and *b* = 3.54, *SE* = 3.45, *p* = .077, 95% CI [−1.56, 12.11], for initiation and inhibition conflicts, respectively. The same pattern was found for “suppression,” with *b* = −0.12, *SE* = 0.52, *p* = .411, 95% CI [−1.12, 0.91] and *b* = 2.77, *SE* = 4.07, *p* = .185, 95% CI [−3.90, 12.43], for initiation and inhibition compared to persistence conflicts. Thus, endorsing these three strategies in situations, in which they demonstrated comparatively higher self-regulatory success, was not significantly associated with higher levels of self-regulatory success in general.

## General Discussion

Given that good self-control is associated with many positive life outcomes, including higher levels of work and academic performance, better physical health, higher well-being, and happier relationships (De Ridder et al., 2011; Hofmann et al., 2014; Moffitt et al., 2011; Tangney et al., 2004), understanding the processes that enable good self-control is highly important for basic and applied psychological research. Here, we have focused on people’s momentary self-regulatory success during self-control conflicts and presented the first study that investigates self-regulatory strategy use as its predictor while differentiating between three different types of self-control conflicts, namely, conflicts that require (1) initiating disliked activity, (2) persisting in disliked activity, or (3) inhibiting an impulse (Carver, 2019). Below we would like to discuss our results but also limitations of our approach.

### Individual Differences in Self-Regulatory Success in Three Types of Self-Control Conflicts

In our study, person differences alone predicted 11.6% of variance in subjective self-regulatory success during self-control conflicts, attesting to important individual differences. In addition, person × conflict type interactions explained an additional 34.3% of variance in subjective self-regulatory success. This means that to understand and predict when people will succeed in managing their self-control conflicts, it is important to take individual differences but also differences between conflict types into account: Whereas some people appear to be better at regulating themselves when confronted with one kind of self-control conflict, other people appear to be better at regulating themselves when confronted with another type of self-control conflict.

This finding is line with an attempt to develop a scale that assesses individual differences in global self-control separately for the three types of self-control conflicts (Hoyle & Davisson, 2016). Even though the authors report relatively strong correlations between the three subscales, they also report separation between them in terms of association with other personality traits. Neuroticism, for example, is more strongly associated with self-control by inhibition than with self-control by initiation. This corresponds to possible predictions based on cybernetic Big Five theory which distinguishes between two independent meta-traits that can reflect covariation between Big Five domains on a superordinate level (DeYoung, 2006; 2010). Stability, the first meta-trait, captures the covariation between (low) neuroticism, agreeableness, and conscientiousness and reflects a tendency towards resisting temptations that would disturb current goal pursuit. Plasticity, the second meta-trait, captures the covariation between openness and extraversion and reflects a tendency to pursue new goals and be flexible with regard to entertaining new perspectives on current goals and strategies. Given conceptual overlaps, we would expect that the stability-related traits (low) neuroticism, agreeableness, and conscientiousness correlate more strongly with self-control during conflicts of inhibition whereas the flexibility-related traits extraversion and openness correlate more strongly with self-control during conflicts of initiation (see Hoyle & Davisson, 2016, for the same argument).

The idea that people’s abilities to resolve the three types of conflict are relatively independent of each other, is also in line with the view that distinct aversive and appetitive motivational systems underlie affective and behavioral response tendencies and dimensions of personality (Carver, Sutton, & Scheier, 2000; Carver & White, 1994; Davidson, 1998; Gable, Reis, & Elliot, 2000; Gray, 1994; Harmon-Jones & Allen, 1997; Lucas, Diener, Grob, Suh, & Shao, 2000; Tellegen, 1985). On the one hand, the behavioral activation (or sometimes approach or facilitation) system (BAS) is responsible for approach and (most types of) positive affect (Carver & White, 1994; Fowles, 1993; Gray, 1994). It is sensitive to rewards and activity in this system causes a person to begin or increase movement towards goals (Carver & White, 1994). On the other hand, the behavioral inhibition system (or sometimes withdrawal) system (BIS) is responsible for inhibition in response to threats and for withdrawal behavior and anxiety (Carver & White, 1994; Davidson, 1992; Fowles, 1993; Gray, 1972, 1994). It is sensitive to punishment and it inhibits behavior that may lead to painful outcomes. Support for the relative independence of these two systems comes from research that has shown their independent neural bases (Gray, 1987a, 1987b; Quay, 1993; Sutton & Davidson, 1997). Moreover, personality differences in healthy adults as well as psychopathologies in clinical populations can be classified according to whether they are accounted for by BAS and BIS sensitivity (e.g., Fowles, 1993; Quay, 1988, 1993). Importantly, the distinction between the BAS and the BIS corresponds with the distinction of initiation/persistence and inhibition: Whereas the BAS should be mostly relevant for initiation and persistence of goal-directed behavior, the BIS should be more relevant to the inhibition of goal-inconsistent behavior. In our data, we also found that self-regulatory success in, on the one hand, initiation and persistence conflicts and, on the other hand, inhibition conflicts were relatively independent, as evidenced by a small association of *r* = .12. Thus, individuals differ with regard to how well they can control themselves during different types of conflict (Hoyle & Davisson, 2016).

### Strategy Choice

Generally, we find that people deploy a large variety of strategies across these three types of self-control conflicts. Our data furthermore showed large differences in the popularity of different self-regulatory strategies. Some strategies were chosen rarely (e.g., “reappraisal”), others frequently (e.g., “focusing on the positive consequences”). When looking at people’s use of the different self-regulatory strategies, the relative contribution of the three sources of variance—person, conflict type, person × conflict type—also varied greatly. This implies that the extent to which each source of variance contributed to the prediction of strategy use, depends on which strategy is considered. If the contribution of person variance component is large, it implies that differences between people, for example, personal preferences, play a large role in predicting whether a strategy is used or not: Some people use a given strategy frequently, others rarely. This is the case, for example, for “focusing on negative consequences” (25.6% variance explained), “taking a substance” (19.8%), and “goal setting” (20.3%). In these examples, but also more generally, the portion of variance that could be attributed to person differences was quite large for strategy choice. It would be interesting to investigate further which person characteristics can explain this large amount of variance between people. One idea is that people take different approaches to goal pursuit and that this is reflected in their strategy choice. On the one hand, there may be the self-controlled way, which is goal-driven and focused and potentially indicated by larger scores on traits like self-control or the conscientiousness facet self-discipline. These persons may prefer strategies that could be described as goal-driven and focused, for example, strategies like “goal setting,” “monitoring progress,” “planning/scheduling,” “putting oneself under pressure,” “reminding themselves of a commitment,” or “suppression.” Others may take a “lighter” approach to goal pursuit, for example, by trying to make it more pleasant or even easier through “seeking social support,” “task enrichment,” or “anticipating self-reward.” To examine this, it might be worthwhile to correlate personality traits like conscientiousness or trait self-control but also individual differences in the quality of motivation (e.g., in controlled versus. autonomous motivation, e.g., Deci & Ryan, 1985; Sheldon & Elliot, 1999) with strategy use to account for the large individual differences between people. It may furthermore be interesting to take a person-centered approach to identify distinct profiles of people who prefer certain groups of strategies over others and link these profiles to these and other individual traits.

Regarding the variance component attributable to the type of self-control conflict only the strategy “distracting oneself from the temptation/activity” stood out, with 10.3% of variance explained by conflict type alone. This implies that whether this strategy was used depended largely on the type of conflict that was experiences. As another analysis showed “distraction” was used most often for conflicts of inhibition.

Lastly, if the contribution of the interaction of person × conflict type variance component is large, as is the case for “changing the environment” (12.2%), “focusing on the positive consequences” (11.4%), and “distraction from the activity/temptation” (11.2%), it implies that whether the strategy is used depends on both the person who is experiencing the self-control conflict and the conflict type in interaction. Or in other words: In these cases, some people prefer using it for one type of conflict, other people prefer using it for other types of conflict. This source of variance is highly interesting, in our perspective, given that our data also show that the subjective efficacy of some strategies—including “changing the environment”—is moderated by conflict type. In a situation where a strategy is clearly more effective for one type of conflict, but people vary in the types of conflict they prefer to use it for, it would make sense to design interventions that teach people who use the strategy for the “wrong” type of conflict to rather use if for the “right” type of conflict. For example, people who use “changing the environment” for conflicts of initiation and persistence, where it does not go along with higher subjective self-regulatory success, could benefit from learning that it is, in fact, only effective for conflicts of inhibition.

### Strategy Efficacy

In terms of the subjective efficacy of other strategies, our analyses of main effects showed that 8 (out of 22) strategies were generally, that is, across the three conflict types, experienced as adaptive and three strategies as maladaptive. Considering conflict type as a moderator, however, revealed more nuance in the relations between strategy use and subjective self-regulatory success. In fact, some strategies, were only experienced as adaptive for certain types of self-control conflicts: “Changing the environment” was only reported as effective for conflicts of inhibition. “Task enrichment” was only reported as effective for conflicts of initiation. “Distracting oneself from the temptation/activity” had no significant effect during conflicts of inhibition and was, in fact, subjectively maladaptive if deployed during disliked activities participants wanted to initiate or persist in. This finding confirms our hypothesis insofar as we had predicted that the subjective effectiveness of distraction would be moderated by conflict type and that it would be negative for conflicts of initiation and persistence (see Hennecke et al., 2019). However, we had also predicted that, in line with previous findings (Mischel et al., 1972), distraction should be perceived as effective during conflicts of inhibition and this part of the hypothesis was not confirmed. Finally, “self-affirmation” and “suppression” had no perceivable beneficial effects during conflicts of persistence but were subjectively effective for conflicts of initiation and conflicts of inhibition.

Together, these results highlight the importance of not repeating, in self-control research, the “fallacy of uniform efficacy” which was previously committed in research on emotion regulation (Bonanno & Burton, 2013) and shows that generalizing results about strategy efficacy from studies looking at only one type of self-control (e.g., persistence conflicts: Hennecke et al., 2019; inhibition conflicts: Friese & Hofmann, 2016; Lopez et al., 2021; Milyavskaya et al., 2021; Williamson & Wilkowski, 2020) to other self-control conflicts or self-control more generally may lead to wrong conclusions. More generally, these results highlight the complexity of self-regulation in the real world.

Which type of self-control conflict is experienced might only be one example for a situational variable that moderates subjective strategy efficacy. As argued above and elsewhere (Hennecke & Bürgler, 2020), additional variables that might interact with each other might complicate deriving clear conclusions about strategy efficacy: Individual trait and state differences (e.g., in self-control, working memory capacity, cognitive load), differences in goals (e.g., between approach and avoidance goals), or other demands of the self-control conflicts (e.g., how difficult it is to resolve, whether a disliked activity is boring, mentally effortful, or physically effortful) might all contribute to the momentary efficacy of a given strategy. However, given the large number of strategies and situational factors, finding ways to examine the complex interplay between strategies and situational factors represents an important challenge for future research.

### The Association of Strategy Choice and Efficacy

The relatively high correlation between the subjective efficacy of strategies and their popularity shows that subjectively effective strategies were also, in general, used more frequently. Given that a correlation does not inform us about the causal direction of this association, we can only speculate about the underlying processes. First, people may choose the strategies they perceive to be more effective more often. This might reflect both inaccurate as well as accurate metacognitive knowledge about the efficacy of self-regulatory strategies for given self-control conflicts (Bürgler et al., 2021). Second, it may be the case that strategies that are more frequently used become more successful, for example, through practice. Third, a third variable, for example, a strategy’s ease of implementation, may affect both its efficacy and its popularity. The current research cannot tell us which of these explanations holds true.

Despite the overall positive correlation between the strategies’ popularity and their subjective efficacy, there are nevertheless a couple of strategies whose popularity diverges from their subjective efficacy. The strategy “focusing on negative consequences,” was popular overall (frequency rank 4) but also relatively ineffective overall (efficacy rank 16). “Self-affirmation” and “task enrichment” were most popular for the conflict types that they were not most effective for. In contrast, the strategy “adopting a process focus” was relatively effective (efficacy rank 8) but unpopular (frequency rank 21). These divergences might reflect misbeliefs about certain strategies. It could be a fruitful endeavor for applied research to correct such misbeliefs and inform people about strategies that are similarly applicable but more effective (e.g., “focusing on the positive consequences” instead). It might also be the case that some effective strategies are used rarely because they can only be implemented under certain conditions. “Adopting a process focus” might, for example, be a strategy that with its attention to how an activity is performed imposes cognitive load and is therefore not applicable when people need to focus on other aspects of the task at hand. The attention of a person studying for an exam might, for example, already be fully absorbed by the contents that need to be understood and memorized, leaving no room for simultaneously focusing on the process of studying itself. During other types of self-control conflicts, for example, when mental load is low, for example, during physical exercise, the strategy might be applicable, nevertheless.

### Strategy-Situation Fit and Individual Differences in Regulatory Flexibility

Our analyses focusing on the moderation of strategy efficacy by conflict type have supported the notion that some strategies are, at least in participants’ experience, more effective for some types of conflict than for others. What these analyses cannot show is whether individuals who use a particular strategy flexibly for the “right” types of conflicts actually benefit from this flexible usage, that is, by experiencing greater self-regulatory success overall. This ability to create strategy-situation-fit, should, however should be an important aspect of individual differences in regulatory flexibility, and in turn, of individual differences in people’s general self-regulatory competence. A similar idea was already proposed for emotion regulation, where three aspects of individual differences in regulatory flexibility were proposed: (1) context sensitivity, (2) strategy repertoire, and (3) feedback monitoring (Bonanno & Burton, 2013). In the self-control domain, there is, as of yet, only evidence that people benefit from a larger repertoire of self-regulatory strategies (Bürgler et al., 2021), from monitoring their use, the situation, and the efficacy of self-regulatory strategies for self-control (Bürgler et al., 2021), and from being more variable in their use of self-regulatory strategies (Wenzel et al., 2021). Evidence for the importance of context sensitivity, which has been defined as “the ability to perceive impinging demands and opportunities from the situation context […] and to determine the most appropriate regulatory strategy in response to those demands and opportunities” (Bonanno & Burton, 2013) was still lacking.

We believe that the current results provide preliminary evidence that this aspect of regulatory flexibility, the ability to select strategies that fit a given situation can, indeed, be an important component of individuals’ self-control: People who used the strategies “changing the environment” and “distraction” more often for conflicts of inhibition conflicts, for which they were found to be more effective than for the other conflict types (for which they were either maladaptive or neither adaptive nor maladaptive), reported higher self-regulatory success overall. These results suggest that regulatory flexibility may be an important aspect of individual differences in self-regulatory competence.

From a theoretical perspective, it would also be desirable to investigate associations of global individual differences in self-control and regulatory flexibility. So far, research has yielded unequivocal results: Feedback monitoring, on the one hand, has been found to correlate positively with trait self-control, implying that it may be one of the processes through which trait self-control “gets outside the skin” (Bürgler et al., 2021). Strategy repertoire, on the other hand, has been found to not be correlated with trait self-control, implying that strategy use may represent a different route to self-regulatory success independent of trait self-control (Bürgler et al., 2021). Given this lack of an association between trait self-control and strategy repertoire, strategy-situation fit may also not be correlated with global individual differences in self-control, given that a large repertoire should be a precondition for people’s ability to create strategy-situation fit (Bonanno & Burton, 2013).

### Limitations and Future Directions

The two studies presented here represent a good start for studying the complexities of self-regulatory strategy use in daily life. The Pilot Study relied, with the DRM, on a bottom-up approach and was able to produce a list of self-regulatory strategies on which various coders could agree and that likely reflects the way lay people act when confronted with self-control conflicts in their daily lives. With its focus on within-person effects, this study took the notion seriously that hypotheses about intraindividual processes should be investigated as such, that is, in within-person analyses (Molenaar & Campbell, 2009). Despite these methodological advantages of the current work, both studies are not without limitations.

The potentially largest limitation of this work might have resulted directly from our ambition to capture the complexity of self-regulation “in the wild.” Even with our sample of 14,000 reported daily self-control conflicts, the evidence for the effectiveness of some strategies for some types of self-control conflicts should be considered preliminary. This is the case because some of the 22 different strategies were reported rarely for some type of conflicts. In turn, their efficacy is estimated based on relatively few observations. At the same time, we wonder what feasible replication studies could look like, given that larger samples may be difficult to attain for these types of studies that are burdensome for participants and therefore expensive for researchers who need to provide appropriate reimbursement. We do not think that, to reduce complexity, simply reducing the number of different strategies participants can choose from is advisable. Assessments with fewer strategies to choose from would either have to lump together different strategies that do not reliably correlate with each other and that do not perform equally well (e.g., “focusing on the positive consequences” and “focusing on the negative consequences” are both forms of reappraisal but only “focusing on the positive consequences” appears beneficial for self-control, see Hennecke et al., 2019; Wenzel et al., 2022) or refrain from assessing the full range of strategies that participants actually use “in the wild.” In our perspective, both options would not do the complexity of self-regulation in everyday life justice. And this complexity is probably even larger than our study suggests. In fact, self-control conflicts vary on many more dimensions than the distinction between initiation, persistence, and inhibition. Initiation and persistence conflicts, for example, may refer to activities with very different aversive demands, including boring activities, physically effortful activities, or cognitively effortful activities (Hennecke et al., 2019). Further complicating the issue, many of the strategies investigated can be implemented in different ways. For example, task enrichment during an aversive task can be achieved through music, TV, phone talk, eating, drinking, etc. It is possible that certain implementations of the same strategy are generally more effective than others, or it is possible that certain implementations can be used across different types of conflicts (or, more generally, situations) with similar success, while others, such as watching TV, might be more limited in where they can be implemented effectively. Even though this study already provides a rather detailed account of the different self-regulatory strategies people can use, it is possible that future studies would benefit from an even more nuanced differentiation of the self-regulatory strategies or “tactics” through which strategies can be implemented (McRae et al., 2012). We hope that future research does not shy away from investigating such complex strategy-by-situation-interactions even though progress may be incremental, and findings may require cross-validation.

In addition, both studies rely on self-reports which come with a couple of caveats. While the DRM has good ecological validity for assessing people’s activities and experiences in other areas of research (Kahneman et al., 2004), its validity for studying processes of self-control is unknown. We nevertheless hope that given its heavy reliance on internally produced structural reminders of the previous day, participants’ memories are more accurate than when asked to report their self-regulatory strategies more globally. Likewise, whereas experience sampling is by now a very common method for studying self-control (e.g., Converse et al., 2019; Hennecke et al., 2019; Hofmann et al., 2012; Milyavskaya et al., 2021; Williamson & Wilkowski, 2020), its validity for assessing self-control in daily life is not entirely clear either. To date, there has been no cross-validation of results from experience sampling through direct observation. In the case of our own assessments, this means that ultimately, we do not know for sure whether people’s self-reported strategy use and self-regulatory success would converge with objective assessments. One danger is that people who believe that a given strategy is successful, even if this is a misbelief, may report that they regulated themselves more successfully, even if they did not. A desire for consistency may cause such biased responding (Festinger, 1957).

Nevertheless, there are good reasons why we used these self-report measures. First, many strategies are intrapsychic strategies, which cannot easily be observed from the outside we studied, among them the five most popular strategies “focusing on positive consequences,” “suppression,” “reminding oneself of a commitment to oneself or to others,” “focusing on negative consequences,” and “thinking of the near finish.” Second, we were determined to study a wide variety of self-control conflicts, including the three types but also, within these types, conflicts that entailed various temptations and activities. Assessing self-regulatory success across a great variety of different self-control conflicts and many participants does not seem feasible with more objective measures. This is not to say that future studies should not extend the current work by, for example, zooming in on specific strategies that can be observed directly, for example, “task enrichment,” and in combination with objective indicators of success.

Another limitation of our assessment lies in the fact that we assessed strategy use with single items rather than more comprehensive scales in the main study. Even if single-item measures are not necessarily worse than multiple-item measures (Bergkvist & Rossitr, 2007) when constructs are quite narrow (Sackett & Larson, 1990), we cannot be sure that we have assessed strategy use with high reliability and with each item fully representing its underlying construct. Single-item measures are, however, very common in experience sampling studies, where otherwise the participant burden would be too high (Friese & Hofmann, 2016; Hofmann et al., 2012; Milyavskaya et al., 2021).

Both methods, the DRM and experience sampling, are furthermore restricted to assessing conscious experiences, here: the conscious deployment of strategies during consciously experienced self-control conflicts. Generally, people may not always be able to accurately report about their intrapsychic processes and it is not clear whether strategy deployment is an exception (Nisbett & Wilson, 1977). Accordingly, both studies did not assess and evaluate strategic attempts that work unconsciously, without being noticed and remembered by participants. Such unconscious regulation has been shown to be effective in helping people to self-control their behavior (Fishbach et al., 2003) but cannot be captured with our method. It is therefore restricted to the assessment of conscious self-regulation.

Finally, we only asked participants to name or choose strategies they used during self-control conflicts that they experienced. Hence, our method did not capture strategies that people use to select of modify situations in a way that prevents self-control conflicts from occurring in the first place (Duckworth et al., 2016; Ent et al., 2015). Some of the strategies we investigated were furthermore used rarely, at least for some type of conflicts, thereby reducing the power for our analyses. This is reflected in the sometimes relatively large confidence intervals around the effect sizes in Figure 2. It would require an even larger sample or a longer period of data collection to gather more data on the effectiveness of rarely used strategies.

## Conclusion

Despite these limitations, the current research provides some important new insights into how self-regulation in the face of daily self-control conflicts works. It highlights the role of self-regulatory strategies and the importance of distinguishing between different types of self-control conflicts, namely, conflicts of initiation, persistence, and inhibition. Finally, we found evidence for the importance of creating strategy-situation fit as an indicator of regulatory flexibility. In conclusion, we want to advocate that future research in the domain of self-control should more strongly consider the complexities of self-control in daily life, if resources permit it. Besides a great variety in the strategies that people can deploy for self-control, there may be a large number of factors, including individual differences and situational features, that determine when a given self-regulatory effort is successful or not.

## Note

1 There was a lot of variety in the situational descriptions of these “other situations” that does not allow us to clearly identify a potential “fourth type” of self-control conflicts. Some self-control conflicts seem like they could have been identified as either one or more than one of the three presented type of conflicts (e.g., “exercise” and “aversion against large supermarkets” could reflect difficulties with initiating or persisting in exercising or shopping, respectively). Maybe because participants faced various conflicts at a time, they were unable to decide for one of the three types of conflicts. In addition, participants’ descriptions of these “other situations” often referred to difficult decisions (“I needed to decide what plant to purchase,” “decide, time pressure, responsibility,” “I needed to decide what to do with my time, which I now had for myself. I needed to decide if I wanted to go for a walk or go home to relax.”).
